# R-Type Calcium Channels Are Crucial for Semaphorin 3A–Induced DRG Axon Growth Cone Collapse

**DOI:** 10.1371/journal.pone.0102357

**Published:** 2014-07-17

**Authors:** Rimantas Treinys, Andrius Kaselis, Emmanuel Jover, Dominique Bagnard, Saulius Šatkauskas

**Affiliations:** 1 Biophysical Research Group, Biology department, Vytautas Magnus University, Kaunas, Lithuania; 2 Institute of Cardiology, Lithuanian University of Health Sciences, Kaunas, Lithuania; 3 INCI – UPR-CNRS 3212, Neurotransmission et sécrétion neuroendocrine, Strasbourg, France; 4 INSERM U1109, MN3t lab, Labex Medalis, University of Strasbourg, Strasbourg, France; Université de Technologie de Compiègne, France

## Abstract

Semaphorin 3A (Sema3A) is a secreted protein involved in axon path-finding during nervous system development. Calcium signaling plays an important role during axonal growth in response to different guidance cues; however it remains unclear whether this is also the case for Sema3A. In this study we used intracellular calcium imaging to figure out whether Sema3A-induced growth cone collapse is a Ca^2+^ dependent process. Intracellular Ca^2+^ imaging results using Fura-2 AM showed Ca^2+^ increase in E15 mice dorsal root ganglia neurons upon Sema3A treatment. Consequently we analyzed Sema3A effect on growth cones after blocking or modifying intracellular and extracellular Ca^2+^ channels that are expressed in E15 mouse embryos. Our results demonstrate that Sema3A increased growth cone collapse rate is blocked by the non-selective R- and T- type Ca^2+^ channel blocker NiCl_2_ and by the selective R-type Ca^2+^ channel blocker SNX482_._ These Ca^2+^ channel blockers consistently decreased the Sema3A-induced intracellular Ca^2+^ concentration elevation. Overall, our results demonstrate that Sema3A-induced growth cone collapses are intimately related with increase in intracellular calcium concentration mediated by R-type calcium channels.

## Introduction

Semaphorin 3A (Sema3A) is one of the key molecules in axon pathfinding repulsing axons during development and inhibiting successful regeneration after injuries of both central and peripheral nervous systems [1], [2]. Since the discovery of chick collapsin [3], and its mammalian ortholog-Semaphorin III/D [4], debate about the significance of Ca^2+^ ions in Semaphorin signal transduction during neural development has evolved. Initial studies, suggesting that Ca^2+^ plays a role in neurite outgrowth, were made before the discovery of guidance factors [5] and early hypothesis claimed that guidance cues should act through fluctuations of intracellular calcium concentration ([Ca^2+^]_i_) optimal levels in axons [6]. Although several groups opposed to that work [7], [8] it is still widely accepted, that intracellular Ca^2+^ coming from internal Ca^2+^ stores is an important part of the response to attractive guidance cues. Recent findings support the idea that Ca^2+^ can act as a messenger in axon growth cone motility and exert an influence on bidirectional axon growth cone turning [9], [10]. Furthermore, the uneven distribution of [Ca^2+^]_i_ in the growth cone and the source of Ca^2+^ are both important for the proper response of the growth cone [9]. Current hypothesis states that Ca^2+^ influx through voltage gated calcium channels (VGCC) mediates growth cone repulsion [11]; and that the elevation of [Ca^2+^]_i_ due to the release from the endoplasmic reticulum (ER) causes attraction [12], [13]. Different calcium ion levels with regard to the neuronal response to guidance factors have been observed between species, and also between types of neurons of the same organism [10]. The similar is true for the expression patterns of calcium channels [14]–[16]. Moreover, several studies showed that expression patterns of Semaphorin 3A (Sema3A) undergo dramatic changes during mouse embryogenesis, reaching its maximum at E15.5 [17], [18]. In this study we aimed to evaluate the role of calcium in Sema3A induced E15 mice dorsal root ganglia (DRG) axon responses and to identify what type of calcium channels can be involved in these responses. To this end we performed general and specific calcium channel inhibition using various VGCC inhibitors: 1) cadmium, known as a non-specific blocker of L, N, P, Q, R, T-type calcium channels [19]–[23], 2) nifedipine (Nif), a selective L-type calcium channel blocker [24], [25], 3) NiCl_2_ – an agent affecting activity of T and R type calcium channels [26]–[30] and 4) SNX482-a specific R-type calcium channel inhibitor [31], [32]. To fully understand if Ca^2+^ channels inhibition is specifically linked to Sema3A-induced DRG axon collapse, this systematic pharmacological approach was performed in parallel with intracellular Ca^2+^ imaging in the presence of Ca^2+^ sensitive dye Fura-2 AM.

## Materials and Methods

### Preparation of explants

C57B1/6J mice were held and maintained according to the Guide for the Care and Use of Laboratory Animals (Lithuanian food and veterinary service permission to work with laboratory animals number B1-287). The experimental protocol was performed in accordance with the national guidelines and approved by State Food and Veterinary Service, Vilnius, Lithuania (Permit numbers: B1-297 and 0236). All efforts were made to minimize suffering. Fifteen days pregnant female mice were sacrificed by cervical dislocation. In order to avoid any possible intermingle between intracellular calcium concentration changes during neurite maturation and growth cone collapses in response to Sema3A as an object for our study we have chosen pseudo-unipolar dorsal root ganglia (DRG) neurons that possess only axons. DRG were dissected from entire vertebral column of fifteen days old embryos (E15). Excised DRG were maintained in ice cold HBSS (Gibco) supplemented with 6.5 mg/mL glucose. DRG were placed on 24×24 mm glass cover slips pretreated by boiling in pure ethanol and thereafter coated with poly-L-lysine (0.01 mg/mL, Sigma) and laminine (0.01 mg/mL, Sigma). Cover slips with DRG were placed in 35 mm Petri dishes filled with 2 mL of Neurobasal (Gibco) growth media supplemented with 2% B-27 (Invitrogen), 100 ng/mL nerve growth factor (NGF, Invitrogen), 5% of fetal bovine serum (FBS, Gibco), 100 U/mL Penicillin (Invitrogen), 100 ng/mL Streptomycin (Gibco) and 4 mg/mL methyl-cellulose (Sigma). DRG’s were grown in humidified 5% CO_2_ atmosphere at 37°C.

### Sema3A purification

Human embryonic kidney 293 (HEK293) cells (CRL 1573; American Type Culture Collection, Manassas, VA) stably transfected with an expression vector containing cDNA coding for Flag-His-Sema3A [17] (cell line 602.108), used as a source of Sema3A, were cultured in minimal essential medium containing 50 U/mL penicillin, 50 µg/mL streptomycin, 500 µM L-glutamine, 10% FCS, and 1 mg/mL G418 (Life Technologies). Sema3A was purified using an anti-Flag M2 affinity gel (Sigma), and its protein concentration was determined using the Bradford method. Concentration of Sema3A as high as 100 ng/mL, if not stated otherwise was used in all experiments.

### Calcium signal modifiers

For blocking of different Ca^2+^ channels and pumps: 1 µM CdCl_2_ for L, P/Q, N, R and T type, 50 nM SNX482 for R type, 10 µM nifedipine (Nif) for L type (all from Sigma) and 100 µM NiCl_2_ for R, T type (from Roth) plasma membrane Ca^2+^ channel; 1 µM thapsigargin (Thap) for Serca 1; 2; 3 (Sigma) were used. To ensure full effect of the Ca^2+^ channel modifiers, DRG with these modifiers were incubated for 60 min.

### Growth cone collapse assay

**Figure 1:**
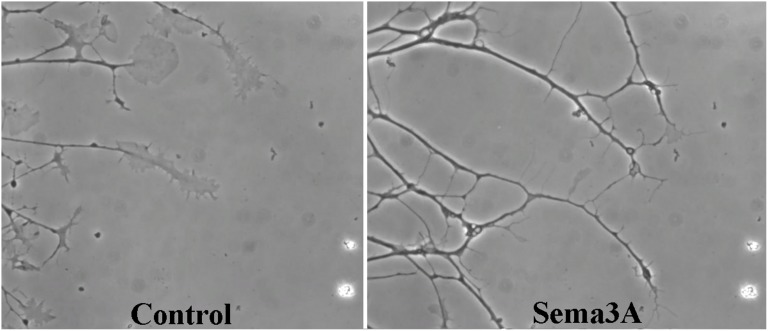
Growth cone morphology in control conditions and after application of Sema3A. Same region of time-laps microscopy represents typical morphology change of growth cones grown for 23 hours in control conditions and after 60 minutes incubation with 100 ng/mL of Sema3A.

For testing of growth cone collapse response to Sema3A, DRG after plating were grown in Neurobasal growth medium at humidified 5% CO_2_ incubator at 37°C for 23 h and then treated with 100 ng/mL Sema3A and/or specific drug. To be consistent with the collapse rate evaluation in response to Ca^2+^ channel modifiers collapse rate was evaluated 60 min after Sema3A addition. A specific Ca^2+^ channel modifier was applied 1–2 minutes in advance of Sema3A treatment. After experimental procedures DRG were fixed in 2% formaldehyde (Roth) solution in growth medium for 10 min following 4% formaldehyde fixation in PBS for 10 min at room temperature. Fixed DRG then were washed with distilled water and then placed in Petri dish filled with 2 mL PBS. All DRG were photographed by using inverted microscope Nicon Eclipse TS100 (Japan) equipped with Motic Moticam 2000 digital camera. Growth cones were evaluated as collapsed according to Fan et al. [33]. Shortly growth cones possessing no lamellipodia and not more than two fillopodia were scored as collapsed, transient stages of growth cones were excluded from evaluation, typical collapsed and intact growth cones are presented in Fig. 1. All single identifiable growth cones were scored and at least 20 growth cones per one DRG were evaluated. Bar graphs for growth cone collapses represent means of at least 160 DRG axon growth cones evaluated obtained in at least three independent experiments.

### Gene array profiling

For gene array profiling DRG were grown at the same conditions as for the growth cone collapse assay. RNA was extracted from DRG explants growing 24 hours in control conditions or in conditions with 100 ng/mL Sema3A using RNA extraction kit (Qiagen, RNeasy mini kit). Quantity and quality of RNA were determined by spectrometry (Thermo Scientific, Nanodrop) and by electrophoresis in 2% agarose gel. Gene array profiling was performed by IGBMC (Strasbourg) using Affimetrix platform. After data processing at IGBMC, it was assumed that gene expression of a specific gene was present if the signal strength for that gene was higher than 4.

### Intracellular Ca^2+^ imaging

Intracellular [Ca^2+^]_i_ recordings were performed on DRG neurons loaded with 10 µM Fura-2 AM-0.04% Pluronic 127 for 30 min in humidified 5% CO_2_ incubator at 37°C, washed twice in HBSS and incubated for 30 more min. The cells were then transferred to an inverted epifluorescence microscope (Axiovert, Zeiss, Germany) equipped with an UPlanFL 40/0.75 objective. The cells were alternatively illuminated at 340 nm and 380 nm and image pairs of the 520 nm light emission were recorded every 2 s for 30 min. Recordings were followed using MetaFluor Fluorescence Ratio Imaging software. The ratio of fluorescence intensities (Ex340 nm/Ex380 nm) was calculated on a pixel basis for each image pair. Increase in relative Fura-2 340/380 fluorescence indicates increase in free intracellular calcium [34], [35]. Appropriate regions of interest (ROI), representing DRG neuron somas, axonal shafts and growth cones were hand selected and average ratio of fluorescence is represented in graphs.

### Statistical analysis

**Figure 2:**
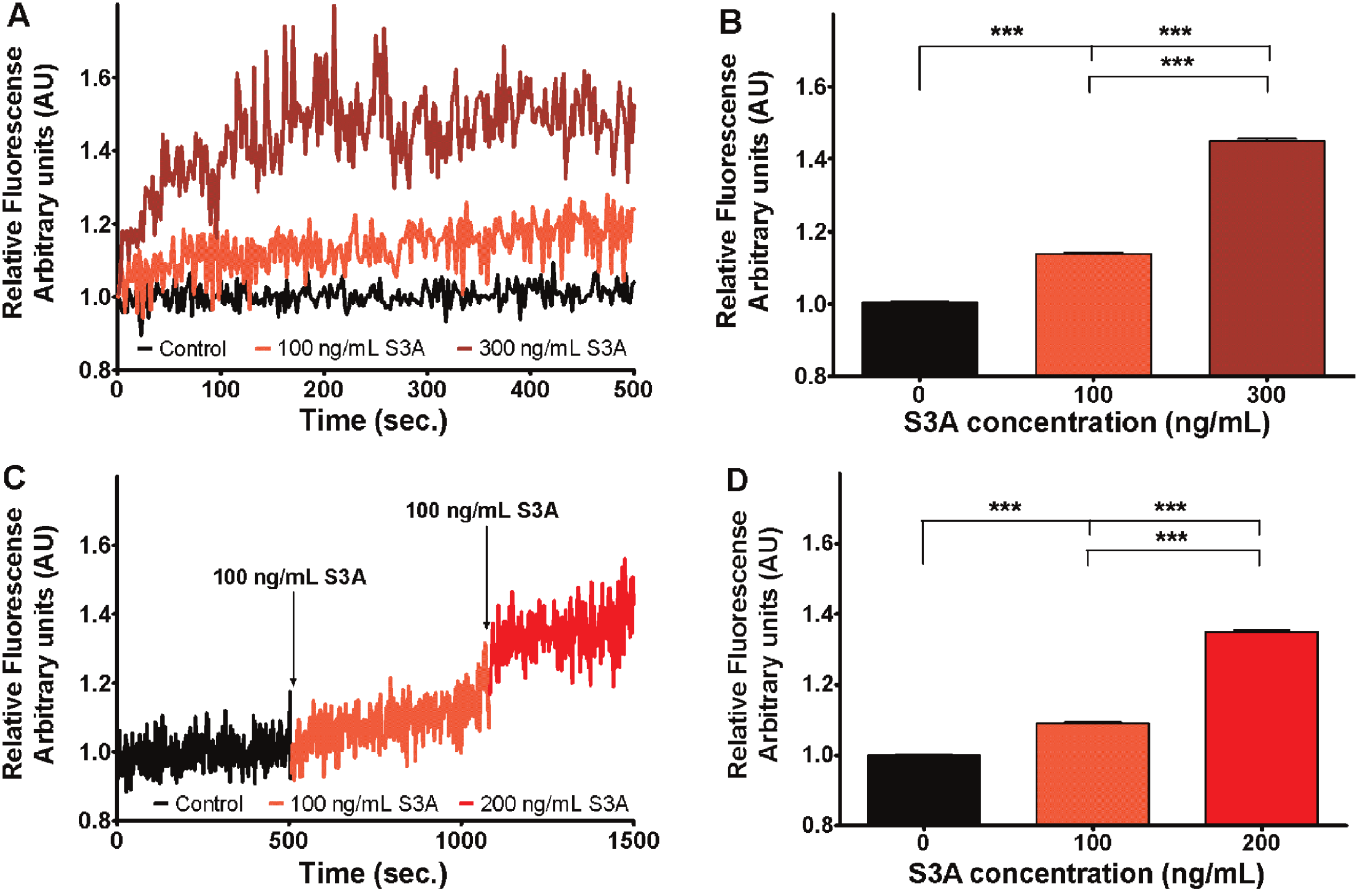
Increase of Sema3A induced Fura-2 fluorescence 340/380 ratio, corresponding to increased [Ca^2+^]_i_ concentration in growth cones. Two different protocols are shown. In panel A, different dishes were used to evaluate the Sema3A concentration dependent effect, while in panel C, the effect of addition of supplemental 100/mL Sema3A concentration was measured recording the same ROIs. All data have been normalized to the fluorescent ratio at the time point preceding Sema3A addition (the value of 340/380 ratio at this point equals to 1 arbitrary unit AU). A) black curve indicates the relative fluorescence before the addition of Sema3A at indicated concentrations (light red curve and dark red curve). B) Bars represent the mean ± SEM of 340/380 ratio in presence of different Sema3A concentrations. C) Curve indicates relative fluorescence in control condition before addition of 100 ng/mL Sema3A following by addition of 100 ng/mL Sema3A (final concentration 200 ng/mL) at indicated time points. D) Bars represent the mean ± SEM of 340/380 ratio in presence of different Sema3A concentrations. To reveal statistical significance Dunn’s post hoc test for Kruskal-Wallis analysis was performed. In the panels B and D upper and lower lines represent statistical difference of the corresponding groups from control and from 100 ng/mL Sema3A respectively. Here *** denotes p<0.001.

**Figure 3:**
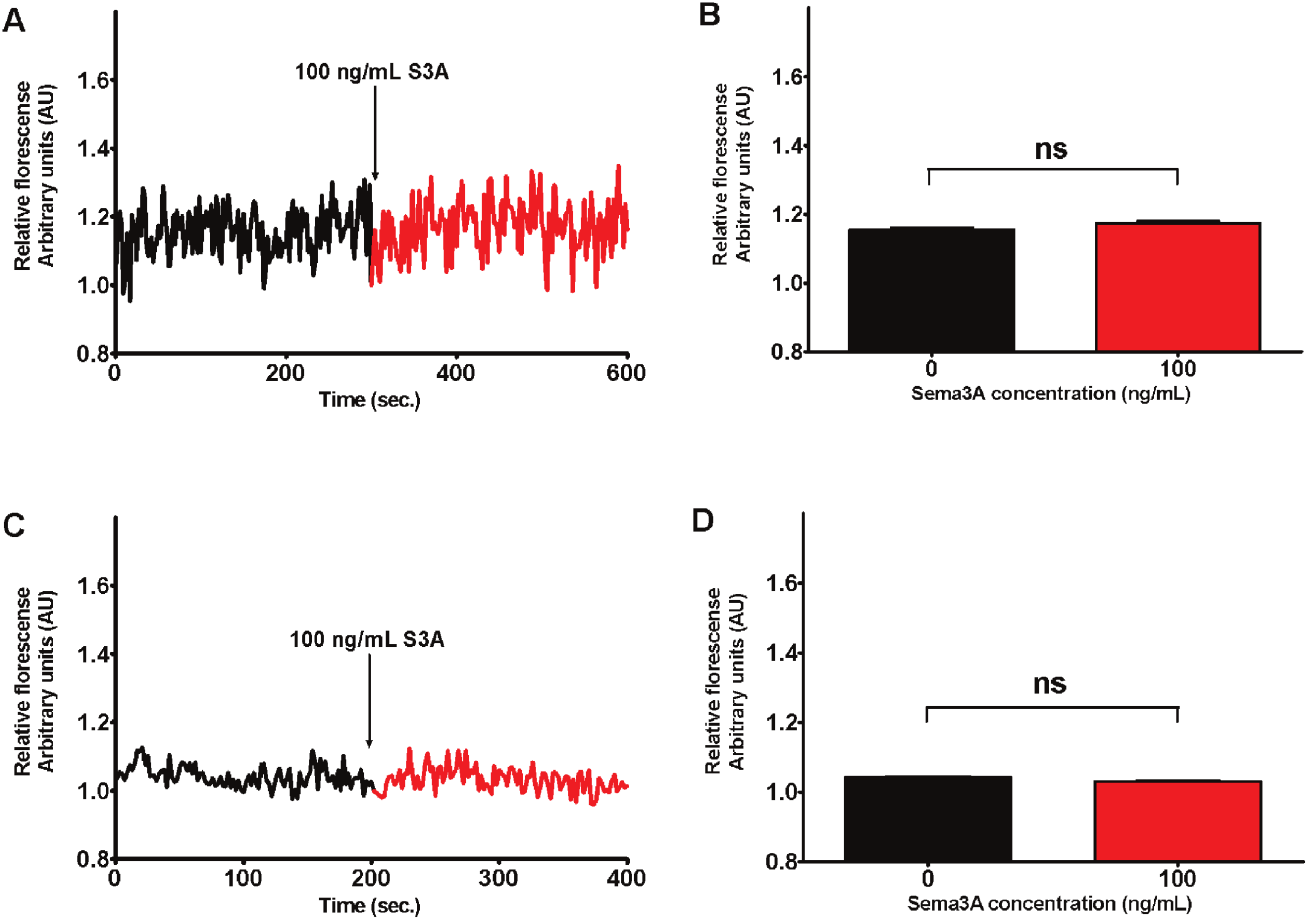
Change of Sema3A (100 ng/mL) induced Fura-2 fluorescence 340/380 ratio, corresponding to change of [Ca^2+^]_i_ concentration in different parts of sensory neurons. Effect of addition of Sema3A concentration was measured recording the same ROIs corresponding to either axon parts proximal to growth cones (panels A and B) or neuron soma (panels C and D). All data have been normalized to the fluorescent ratio at the time point preceding Sema3A addition (the value of 340/380 ratio at this point equals to 1 arbitrary unit AU). A) Curve indicates the relative fluorescence of ROIs of axons (black curve) and after (red curve) the addition of Sema3A. B) Bars represent the mean ± SEM of 340/380 ratio in axons in absence (black bar) and presence (red bar) of Sema3A. C) Curve indicates the relative fluorescence of ROIs of neuron soma before (black curve) and after (red curve) the addition of 100 ng/mL of Sema3A. D) Bars represent the mean ± SEM of 340/380 ratio in neuron soma in (black bar) and presence (red bar) of Sema3A. To reveal statistical significance Dunn’s post hoc test for Kruskal-Wallis analysis was performed. In the panels B and D lines represent statistical difference of control and Sema3A of the same ROIs evaluated. Here “ns” denotes not significant.

**Figure 4:**
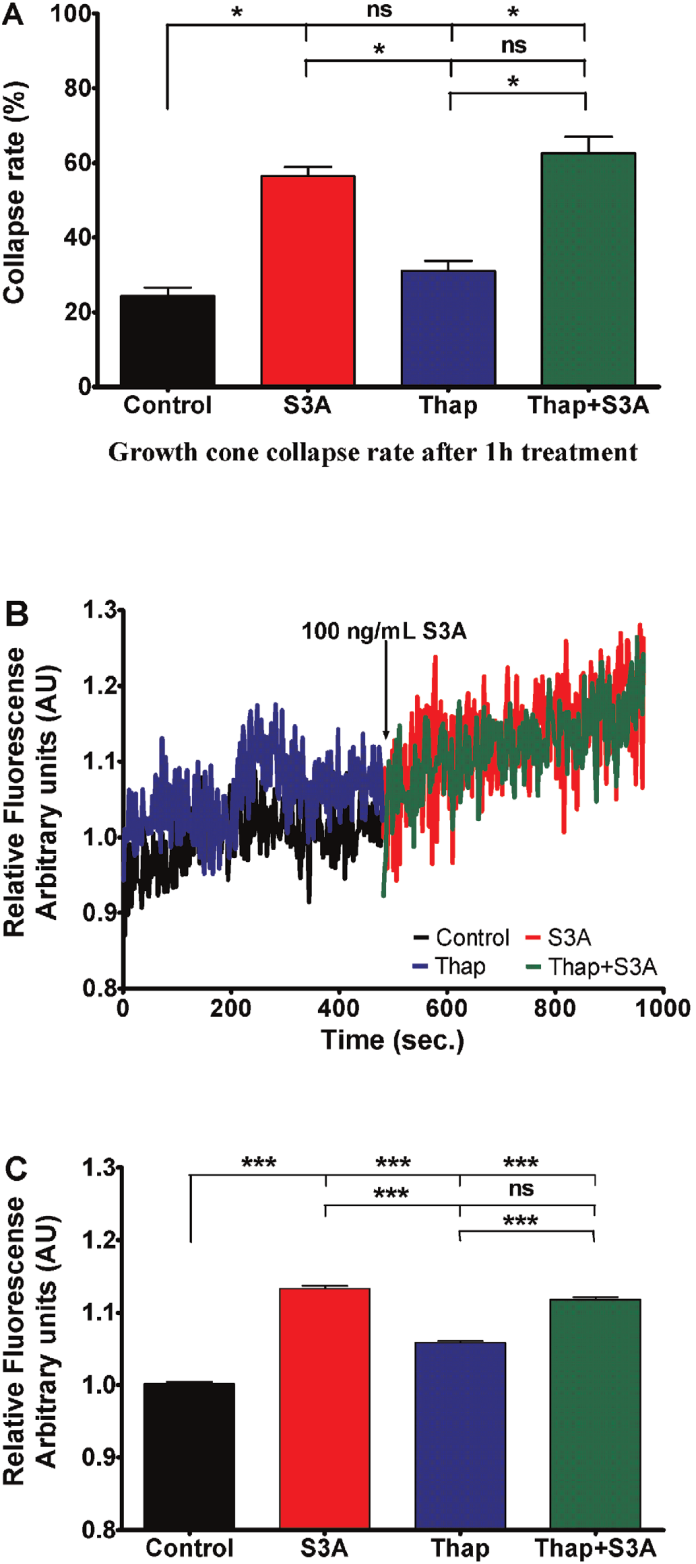
Influence of SERCA inhibitor thapsigargin (1 µM) on Sema3A (100 ng/mL) induced effects. A) 1 hour incubation with endoplasmic reticulum SERCA inhibitor had no significant effect on growth cones of mouse E15 DRG growth cone collapse rate (red bar) comparing to control conditions (black bar). Also, thapsigargin along with Sema3A (green bar) did not change Sema3A collapse-inducing effect (blue bar). B) Changes of relative Fura-2 340/380 fluorescence in control conditions (black curve) and 1 µM of thapsigargin (blue curve) followed by DRG neuron treatment with Sema3A (red and green curves respectively) at indicated time point. All data have been normalized to the point preceding Sema3A addition (this point equals to 1 arbitrary unit AU). C) Bars represent fluorescence mean ± SEM: black bar represents control Fura-2 fluorescence; red bar represents mean fluorescence in presence of Sema3A; blue bar indicates condition in presence of thapsigargin and green bar indicates conditions where Sema3A was added in presence of thapsigargin. Here S3A and Thap corresponds to Sema3A and thapsigargin respectively; ***p<0.001; *p<0.05; ns – not significant. In the panels A and C upper, middle and lower lines represent statistical difference of the corresponding groups from control, from Sema3A, and thapsigargin groups respectively.

**Figure 5:**
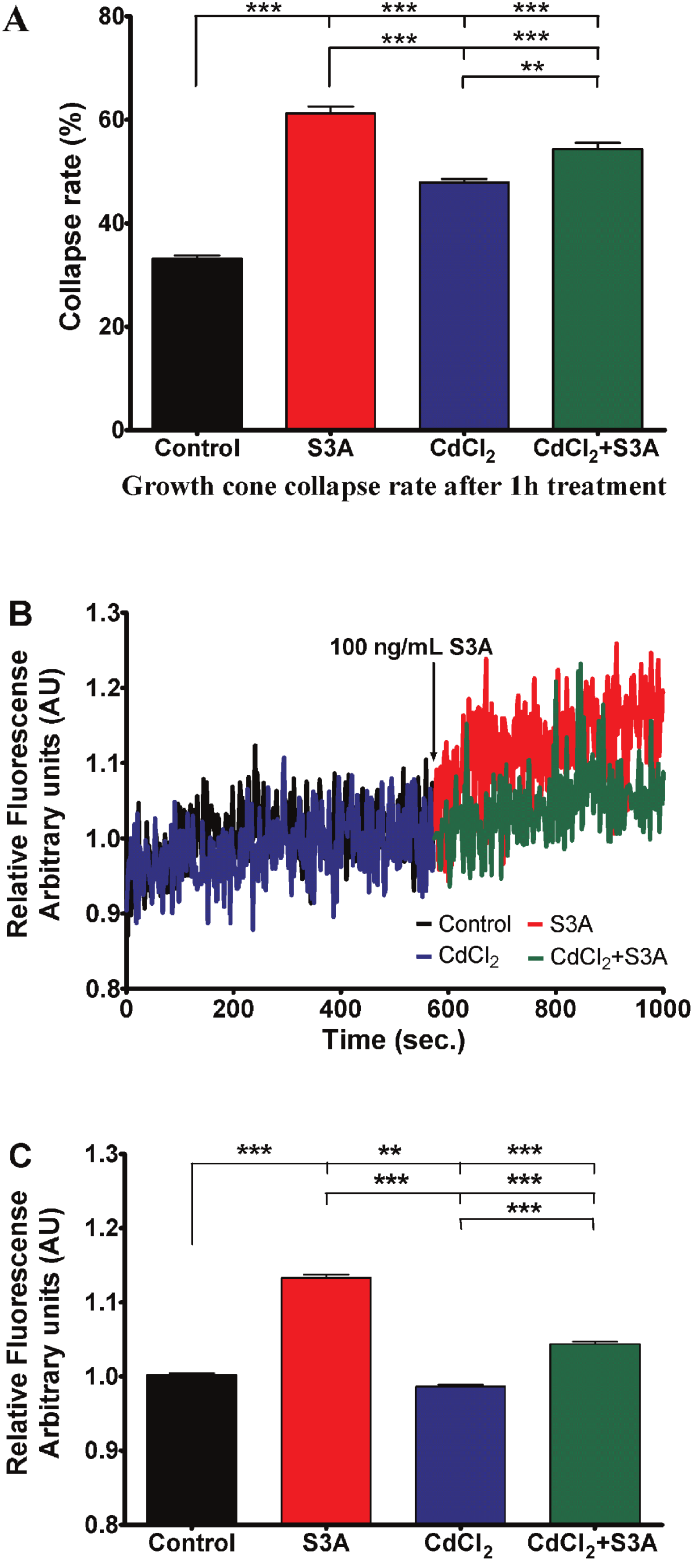
Influence of CdCl_2_ (1 µM), an unspecific blocker of HVA and LVA VGCC on growth cone collapse rate and changes in Sema3A (100 ng/mL) induced intracellular calcium concentration. A) Growth cone collapse rate in control group (black bar) Sema3A treated group (red bar), CdCl_2_ treated group (blue bar) or both treatments (green bar). B) Changes of relative Fura-2 340/380 fluorescence in control conditions (black curve) and 1 µM of CdCl_2_ (blue curve) followed by DRG neuron treatment with Sema3A (red and green curves respectively) at indicated time point. All data have been normalized to the point, before Sema3A was added (this point equals to 1 arbitrary unit AU). C) Bars represent mean ± SEM of relative Fura2 fluorescence: black bar represent control Fura-2 fluorescence; red bar represent mean fluorescence in presence of Sema3A; blue bar indicates condition in presence of CdCl_2_ and green bar indicates conditions where Sema3A was added in presence of CdCl_2_; ***p<0.001; **p<0.01. In the panels A and C upper, middle and lower lines represent statistical difference of the corresponding groups from control, from Sema3A, and CdCl_2_ groups respectively.

**Figure 6:**
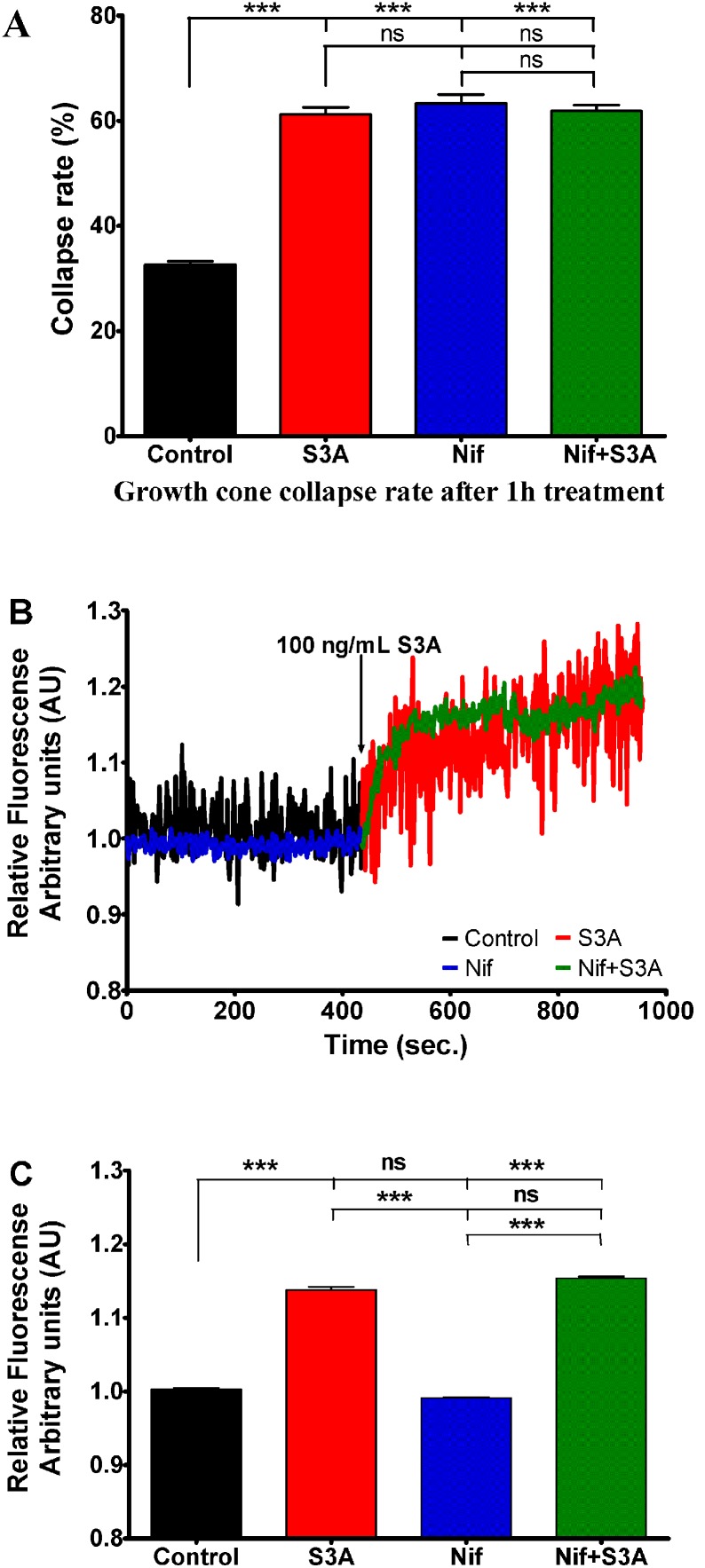
Influence of nifedipine (10 µM), a selective blocker of HVA L-type VGCC on growth cone collapse rate and changes in Sema3A (100 ng/mL) induced intracellular calcium concentration. A) Growth cone collapse rate in control group (black bar) Sema3A treated group (red bar), nifedipine treated group (blue bar) or both treatments (green bar). B) Changes of relative Fura-2 340/380 fluorescence in control conditions (black curve) and 10 µM of nifedipine (blue curve) followed by DRG neuron treatment with Sema3A (red and green curves respectively) at indicated time point. All data have been normalized to the point, before Sema3A was added (this point equals to 1 arbitrary unit AU). C) Bars represent mean ± SEM of relative Fura2 fluorescence: black bar represents control Fura-2 fluorescence; red bar represents mean fluorescence in presence of Sema3A; blue bar indicates condition in presence of nifedipine and green bar indicates conditions where Sema3A was added in presence of nifedipine. Here S3A and Nif corresponds to Sema3A and nifedipine respectively; ***p<0.001; ns–not significant. In the panels A and C upper, middle and lower lines represent statistical difference of the corresponding groups from control, from Sema3A, and nifedipine groups respectively.

**Figure 7:**
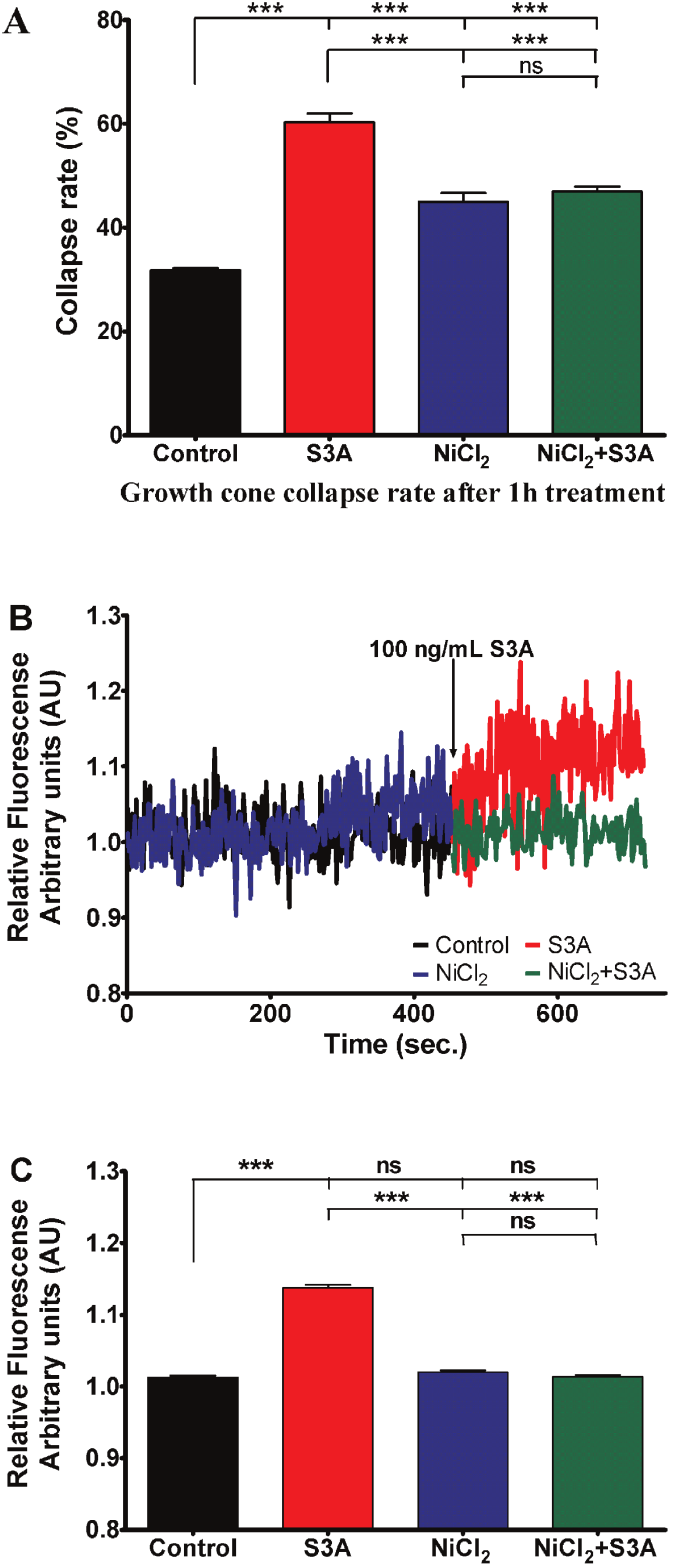
Influence of NiCl_2_ (100 µM), a blocker of T and R type LVA VGCC on growth cone collapse rate and changes in Sema3A (100 ng/mL) induced intracellular calcium concentration. A) Growth cone collapse rate in control group (black bar) Sema3A treated group (red bar), NiCl_2_ treated group (blue bar) or both treatments (green bar). B) Changes of relative Fura-2 340/380 fluorescence in control conditions (black curve) and 100 µM of NiCl_2_ (blue curve) followed by DRG neuron treatment with Sema3A (red and green curves respectively) at indicated time point. All data have been normalized to the point, before Sema3A was added (this point equals to 1 arbitrary unit AU). C) Bars represent mean ± SEM of relative Fura2 fluorescence: black bar represent control Fura-2 fluorescence; red bar represent mean fluorescence in presence of Sema3A; blue bar indicates condition in presence of NiCl_2_ and green bar indicates conditions where Sema3A was added in presence of NiCl_2_; ***p<0.001; ns–not significant. In the panels A and C upper, middle and lower lines represent statistical difference of the corresponding groups from control, from Sema3A, and NiCl_2_ groups respectively.

**Figure 8:**
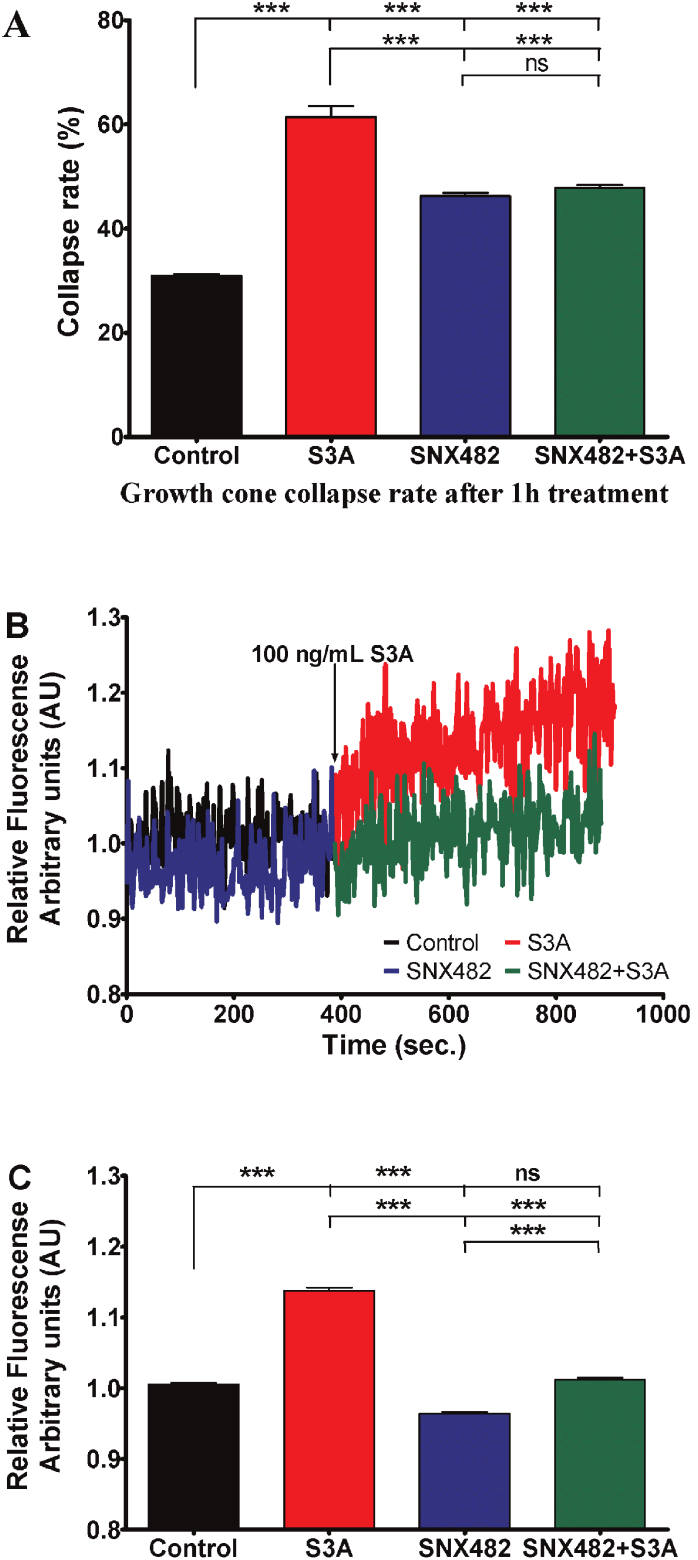
Influence of SNX482 (50 nM), a specific blocker of R type LVA VGCC on growth cone collapse rate and changes in Sema3A (100 ng/mL) induced intracellular calcium concentration. A) Growth cone collapse rate in control group (black bar) Sema3A treated group (red bar), SNX482 treated group (blue bar) or both treatments (green bar). B) Changes of relative Fura-2 340/380 fluorescence in control conditions (black curve) and 50 nM of SNX482 (blue curve) followed by DRG neuron treatment with Sema3A (red and green curves respectively) at indicated time point. All data have been normalized to the point, before Sema3A was added (this point equals to 1 arbitrary unit AU). C) Bars represent mean ± SEM of relative Fura2 fluorescence: black bar represent control Fura-2 fluorescence; red bar represent mean fluorescence in presence of Sema3A; blue bar indicates condition in presence of SNX482 and green bar indicates conditions where Sema3A was added in presence of SNX482; ***p<0.001; ns – not significant. In the panels A and C upper, middle and lower lines represent statistical difference of the corresponding groups from control, from Sema3A, and SNX482 groups respectively.

Results are expressed as mean ± standard error of the mean (SEM) of at least three independent experiments performed. Microsoft Excel 2007 was used to calculate mean for each experimental condition. Statistical analysis and significance of difference between groups was evaluated by two-sided, unpaired Student’s t-test (Table 1; Figures 2; 3; 4A; 5A; 6A; 7A; 8A). Fura-2 intracellular fluorescence analysis was performed using Microsoft Excel for determination of average fluorescence of each experimental condition and GraphPad Prism (version 5 for Windows) for preparation of graphs and statistical analysis. Relative fluorescence ratio was normalized to the point preceding Sema3A addition. The value at this point equals to 1 arbitrary unit (AU). Due to the absence of normal distribution, Kruskal–Wallis nonparametric analysis with Dunn’s post hoc test was used for multiple-group comparisons (Figures 2; 3; 4 B and C; 5 B and C; 6 B and C; 7 B and C; 8 B and C).

**Table 1:** Data from gene expression profiling of E15 mouse DRG’s in two different conditions: control conditions (Control), and bath application of 100 ng/mL Sema3A (Sema3A).

Signal strengthControl	Signal strengthSema3A	Fold changeSema3A/Control	p valueSema3A/Control	GeneSymbol	Type ofchannel/pump
5.29	5.41	1.10	0.11	*Atp2a1*	SERCA (Serca1)
10.69	10.62	0.90	0.21	*Atp2a2*	SERCA (Serca2)
4.86	5.05	1.08	0.23	*Atp2a3*	SERCA (Serca3)
5.23	5.31	1.05	0.51	*Cacna1s*	L-type (Ca_v_1.1)
7.71	7.68	0.91	0.62	*Cacna1c*	L-type (Ca_v_1.2)
6.08	6.32	0.92	0.75	*Cacna1d*	L-type (Ca_v_1.3)
8.70	8.78	1.07	0.18	*Cacna1a*	P/Q-type (Ca_v_2.1)
9.54	9.54	1.00	0.82	*Cacna1b*	N-type (Ca_v_2.2)
8.52	8.57	0.99	0.55	*Cacna1e*	R-type (Ca_v_2.3)
5.27	5.43	1.16	0.45	*Cacna1g*	T-type (Ca_v_3.1)
8.89	8.89	0.97	0.98	*Cacna1h*	T-type (Ca_v_3.2)

Signal strength columns of Control and Sema3A conditions represent average values of three independent experiments. Fold change – fold change of mRNA expression in different conditions, p value – Student’s t-test for significance of difference.

## Results

### Semaphorin 3A induces intracellular Ca^2+^ elevation

Intracellular free Ca^2+^ concentration was measured using the Fura-2 fluorescent probe. In initial experiments we have evaluated growth cone response to 60 mM KCl depolarization, which was used as positive control to check whether change of intracellular Ca^2+^ concentration can be observed at chosen regions of interest (ROI). As 100% of analyzed ROI were sensitive to 60 mM KCl (data not shown), which opens all neuronal VGCC due to electro-osmosis, we concluded that this method of Ca^2+^ imaging is suitable to study whether Sema3A response is a Ca^2+^ dependent process. The Fura-2 signal was recorded at least for 300 s to establish the baseline followed by at least 300 s recording after addition of Sema3A. DRG treatment with 100 ng/mL of Sema3A resulted in slight but significant and steady 13% intracellular Ca^2+^ elevation. When DRG were treated with 300 ng/mL Sema3A concentration the mean fluorescence increased by 42% (from 1.03 AU in control to 1.48 AU in Sema3A condition) (Fig. 2A and 2B). When after DRG treatment with 100 ng/mL with Sema3A an additional 100 ng/mL of Sema3A was added (reaching final concentration of 200 ng/mL) we observed an additional increase by 35% in relative fluorescence compared to control (Fig. 2C and 2D). Overall, these results demonstrate that Sema3A induces a dose dependent elevation of intracellular Ca^2+^ concentration. Strikingly, this part of the experiment showed that Sema3A-induced increase in [Ca^2+^]_i_ concentration in growth cones reaches a plateau setting cytosolic [Ca^2+^]_i_ a new steady level, which can be further increased by supplementary addition of Sema3A.

A similar set of experiments was performed to evaluate whether Sema3A can induce [Ca^2+^]_i_ increase in DRG neuron soma or axon segment close to growth cone. Our results demonstrated, that there is no significant difference in relative Fura2 fluorescence change in response to 100 ng/mL Sema3A neither in neuron soma, nor in axon parts proximal to growth cones (Fig. 3).

Since results showed, that Sema3A dependent Ca^2+^ increase in growth cones is prolonged and sustained event, we performed Ca^2+^ channel gene expression analysis to determine whether Ca^2+^ concentration can be dependent on Ca^2+^ channel expression *de novo*.

### Sema3A sensitive DRG neurons express VGCC and SERCA

In order to ascertain origin of Ca^2+^ mobilized during Sema3A induced increase in [Ca^2+^]_i_ concentration, we first used gene array profiling data that was generated to compare gene expression in control DRG with that in DRG treated with Sema3A at 100 ng/mL concentration. Gene array profiling data showed that in all conditions analyzed, the voltage gated Ca^2+^ channels (VGCC) and SERCA subtypes were expressed in E15 mouse DRG neurons (Table 1). We also found that expression of the channel’s proteins even during prolonged incubation of 24 hours was not significantly modified by the presence of Sema3A.

### Sema3A-induced growth cone collapse and calcium signaling are internal calcium stores independent

To determine whether Sema3A-induced growth cone collapse is dependent on internal Ca^2+^ stores, the cultured DRG were incubated in the presence of the general SERCA blocker thapsigargin, which can block all three SERCA isoforms [36], [37]. In the presence of thapsigargin 31% of axon growth cones were collapsed, a rate not significantly different (p>0.05) from the 24% collapsed axonal tips in the control group. When DRG were incubated in the presence of Sema3A (100 ng/mL), alone or in the presence of thapsigargin, the rate of collapsed growth cones was not different (56% versus 62% respectively, p>0.05) (Fig. 4A). Consistently, experiments performed with Ca^2+^ sensitive dye Fura-2 revealed that Sema3A induced [Ca^2+^]_i_ elevation was conserved in the presence of thapsigargin in spite of a significant increase of the basal level of calcium signaling observed in the presence of thapsigargin alone. Hence, SERCA is neither implicated in triggering Sema3A-induced growth cone collapse, nor is related with [Ca^2+^]_i_ elevation. (Fig. 4B and 4C). Since thapsigargin blocks all Serca pumps, responsible for elimination of calcium form the cytoplasm, temporal increase in [Ca2^+^]_i_ is seen in the panel B of the figure (blue curve).

### Sema3A-induced growth cone collapse and calcium signaling are VGCC dependent

To reveal if Sema3A induced collapses can be modified by currents through voltage gated calcium channels (VGCC) we performed general calcium channel inhibition with CdCl_2_. In comparison to control conditions, 1 hour incubation of DRG in the media containing 1 µM CdCl_2_ increased basal collapse rate from 33 to 48% (P<0.001) thereby demonstrating a clear impact of VGCC in the control of growth cones integrity (Fig. 5A). Strikingly, a significant decrease (p<0.001) of Sema3A induced collapse rate was observed when DRG were concomitantly treated with Sema3A and 1 µM CdCl_2_. These results indicate that Sema3A induced growth cone collapses can be inhibited by blocking plasma membrane calcium channels by unspecific L, N, P, Q, R, T-type calcium channel inhibitor CdCl_2_ [19]–[23]. Indeed, when measuring [Ca^2+^]_i_ using Fura-2 fluorescence we found a significant decrease of the fluorescent signal in the presence of Sema3A and CdCl_2_ compared to Sema3A alone (1.04 AU versus 1.13 AU respectively, P<0.01). These results suggest a relationship between VGCC activity and Sema3A-induced variation in intracellular calcium concentration. (Fig. 5B and 5C).

Since cadmium inhibits L, N, P, Q, R, T – type calcium channels with different affinity, we decided to evaluate influence of both high voltage activated (HVA) and low voltage activated (LVA) Ca^2+^ channels to find out which of them is/are responsible for Sema3A- induced effects.

### Sema3A-induced growth cone collapse and calcium signaling are L-type Ca^2+^ channel independent

We performed experiments using selective high voltage activated (HVA) L-type calcium channel blocker nifedipine. Incubation of DRG neurons for 1 hour in the media containing 10 µM of nifedipine resulted in an increase of growth cone collapse rate from 33% up to 63%. This side effect strongly impaired the possibility to analyze the involvement of HVA channel in growth cone collapse because Sema3A treatment induced 61% collapse rate when added without this calcium channel blocker (Fig. 6A). However, Fura-2 fluorescence imaging revealed that nifedipine by itself did not influence intracellular calcium fluctuations. Interestingly, Sema3A induced [Ca^2+^]_i_ elevation in the presence of nifedipine was at the same level as in the absence of nifedipine thereby suggesting that increase in intracellular calcium concentration was not mediated by L-type calcium channels (Fig. 6B and 6C).

Results obtained by Fura2 fluorescence evaluation in axon growth cones let us exclude influence of L-type calcium channels on Sema3A induced growth cone collapses and elevation of [Ca^2+^]_i_ related to induction of collapse.

### Sema3A-induced growth cone collapse and calcium signaling are LVA Ca^2+^ channel dependent

We have further investigated the potential role of low voltage activated (LVA) T- and R- type calcium channels on Sema3A induced effects by modifying their activity with NiCl_2_ [26]–[30]. Similarly to nifedipine, the addition of NiCl_2_ induced a significant increase of basal growth cone collapse rate from 32% in control group up to 45% in medium with NiCl_2_. However, in contrast to nifedipine, this increase was modest and significantly lower than that of 60% induced by Sema3A alone. When DRG were treated with Sema3A in the presence of NiCl_2_ growth cone collapse decreased to 47%, thereby demonstrating that inhibition of NiCl_2_-sensitive T and R calcium channels suppresses Sema3A-induced growth cone collapses. Moreover, the presence of NiCl_2_ abolished Sema3A induced [Ca^2+^]_i_ elevation as demonstrated in calcium imaging experiments (Fig. 7B and 7C). Altogether these experiments revealed that both growth cone collapse and increase in intracellular calcium concentration are mediated by T or R type LVA calcium channels.

### Sema3A induced growth cone collapses and calcium signaling are R-type Ca^2+^ channel dependent

In order to find out which of the two possible calcium channels T or R can account for Sema3A induced effects experiments with SNX482 (a compound that specifically affects only R type calcium channels) were performed [31], [32].

As for nifedipine and NiCl_2_ the addition of SNX482 induced a significant increase of basal growth cone collapse rate from 31% in control group to 46% with SNX482. However, similarly to NiCl_2_, this higher rate of collapse was still significantly lower than the one induced by Sema3A alone, therefore allowing us to conclude on the role of R-type channel in this assay. Indeed, the addition of SNX482 decreased Sema3A-induced collapse rate significantly (p<0.001) from 61 to 48%. Strikingly, when monitoring Fura2 fluorescence in the different experimental conditions we found that SNX482 treatment almost abolished Sema3A-induced [Ca^2+^]_i_ elevation. Taken together, these results identify R-type calcium channel as the major source of Sema3A-induced intracellular calcium increase (Fig. 8B and 8C).

## Discussion

Axon guidance requires controlled intracellular signals in the growth cone [10], [38] which are crucial for efficient axon navigation [39], [40]. Increasing evidences show that axon guidance is indeed related with changes in intracellular calcium concentration. Tojima and colleagues clearly demonstrated that calcium is important for growth cone attraction in chick DRG neurons [11], [41] and several studies showed that intracellular Ca^2+^ stores are important for axon navigation [42]–[44]. It is still under debate whether Semaphorin guidance cues also require calcium signaling to trigger repulsive guidance effects. Recently Plazas et al., (2012) demonstrated, that Ca^2+^ influx to the growth cones of zebrafish motor neuron can be related to the expression of PlexinA3 that was shown to participate in Sema3A induced guidance [45]. This link is interesting and novel, but as was shown in this study is limited only to certain types of neurons, as suppression of Ca^2+^ spiking by hKir2.1 expression led to substantial pathfinding errors in middle and rostral, but not caudal primary motor neurons of zebrafish. Although we have not investigated spiking activity in our study we have demonstrated that Sema3A induced growth cone collapse of E15 mice embryo DRG neurons is a calcium dependent process. It has been previously shown, that calcium channel distribution and expression varies throughout embryogenesis [46]. Our gene array profiling data (Table 1) showed, that all known voltage gated calcium channel (L, N, P/Q, T and R) α subunits, capable of forming functional Ca^2+^ channels [47] are expressed in E15 mouse DRG explants and thus can be important in neurogenesis *in vivo*. Moreover we found that all three subtypes of SERCA are expressed in these neurons that raise the possibility that intracellular Ca^2+^ stores are also important for Sema3A induced effects. Because knockdown of the different calcium channels using for example siRNA strategy is difficult to perform in a systematic way with proper controls we decided to apply a pharmacological strategy of blocking/modifying activity of the calcium channels and pumps expressed in E15 mouse DRG. To this end we used different calcium channel activity modifying compounds that allowed us an almost systematic approach complicated by unexpected side effects of the drugs increasing the basal rate of growth cone collapse in certain cases. Indeed, both intracellular and extracellular Ca^2+^ are extremely important for normal functionality of neurons [48]. Thus the inhibition of voltage gated channel activity by organic (Nifedipine; SNX482) or inorganic (NiCl_2_; CdCl_2_) compounds could have induced growth cone collapses not related to axon guidance, but rather due to changes in protein activity [49]. Moreover, it is known that various calcium channel blockers do induce some side effects to the cells [50] that in our study is seen as slight increase in growth cone collapse rate. On the other hand, excepting nifedipine, the Sema3A induced-growth cone collapse was always significantly higher than the one induced by the inhibitory compounds thereby enabling us to extract the contribution of calcium channels to Sema3A-induced growth cone collapse. To evaluate if intracellular Ca^2+^ stores are important for Sema3A induced growth cone behavior we have monitored Sema3A effects in the presence of the SERCA inhibitor thapsigargin. Our results showed that modification of SERCA activity had no effect on Sema3A induced collapse rate, excluding the refilling of the reticular Ca^2+^ store as significant for Sema3A induced growth cone collapse in E15 DRG neurons. Thapsigargin was however recently shown to activate ORAI channels in DRG neurons [51]. In our case such an activation of ORAI with thapsigargin had no effect on Sema3A induced elevation of [Ca^2+^]_i_. On the other hand, our results showed that CdCl_2_, a general inhibitor of all plasma membrane VGCC did reduce Sema3A induced growth cone collapse rate and related calcium signal. To find out which of the possible plasma membrane calcium channels participate in Sema3A-induced signal formation leading to the growth cone collapse we used different VGCC blockers. At first we have evaluated nifedipine, a specific L-type HVA VGCC blocker. It was claimed by Yamane and colleagues [52] as well as previously reviewed by Wen and Zheng (2006) that L-type Ca^2+^ channels can be important for response to Sema3A in chick DRG neurons [53]. As previously mentioned, we found that nifedipine itself induced growth cone collapse impeding definitive conclusion with regard to the induction of growth cone collapse. However, calcium imaging showed a persistent increase of intracellular calcium when exposing DRG neurons to Sema3A in presence of nifedipine, this finding let us exclude a role of L-type channel in Sema3A-induced [Ca^2+^]_i_ increase in growth cones, suggesting that L-type channels do not contribute to Sema3A induced [Ca^2+^]_i_ elevation and consequently growth cone collapse in mouse DRG axons. This is in agreement with Chi et al., (2009) and Behar et al., (1999) who also showed that L-type Ca^2+^ channels do not contribute to Sema3A-induced growth cone collapse rate [54], [50]. We further evaluated contribution of LVA channels to Sema3A induced growth cone collapses. Large spectrum LVA calcium channel blocker NiCl_2_ significantly reduced Sema3A induced growth cone collapse rate. This inorganic Ca^2+^ channel blocker at concentrations used (100 µM) blocks ∼30% of T-type Ca^2+^ current and has strong influence on R-type calcium channels affecting ∼90% of these VGCC [55], [22]. Higher concentrations of NiCl_2_ cannot be used, as it start affecting not only low voltage activated currents (LVA) but also high voltage activated (HVA) channel behavior [28]. One of the most important finding of our study is that 100 µM NiCl_2_ prevented Sema3A-induced growth collapses. This led us to assume that T-type, R-type or both Ca^2+^ channels are important for Sema3A induced growth cone collapse. There are to our knowledge no selective inhibitors of T-type channels. Thus, to distinguish which of these channels are important for Sema3A induced effect on growth cones we used the only known specific R-type Ca^2+^ channel blocker SNX482 [56], [31], [32]. Both growth cone collapse assay and calcium imaging results support the finding of a specific role of R-type calcium channel in Sema3A inhibitory signaling because growth cone collapse was fully abolished and most of the calcium signal was gone. This is consistent with recent findings of Nishiyama and colleagues that showed a direct link between Sema3A and R-type Ca^2+^ channels in *Xenopus laevis* commissural interneurons where Sema3A changes axons into dendrites by up-regulating R-type Ca^2+^ channel activity [57]. Moreover Nishiyama et al., (2011) suggested that there should be “X” pathway that modifies expression of R-type Ca^2+^ channels *de novo* and it was recently shown that TRPCs can modify expression of several channel types including VGCC [57]. This may explain how knock down of TRPCs can prevent the effects of guidance cues [58], [59]. This link would further implement that recently discovered involvement of TRPC5 in Sema3A signaling pathway in P1 mouse hippocampus neuron growth cones [60] requires VGCCs. It is further supported by claims of Kaczmarek et al., (2012) that proteases calpain-1 (µ-calpain) and calpain-2 (m-calpain) that were shown to be necessary for Sema3A induced activation of TRPC5 that have the highest expression levels in rodent brain, but not in peripheral nervous system [60]. TRPC are nonselective cation channels and therefore could be responsible for calcium increase following Sema3A application observed in our study. Nevertheless, Kaczmarek et al., (2012) did not discuss the possible source of the initial calcium needed for calpain activation. Therefore the results of our study together with the Kaczmarek et al., (2012) findings [60] suggest that Sema3A can activate R-type calcium channels that in turn, through the increase in intracellular calcium concentration, could contribute to activation of calpains and TRPC. Although the hypothesis is preliminary only, it suggests a plausible link between TRP and R-type calcium channels. Indeed, in support to this hypothesis several studies already showed the link between TRPCs and voltage gated calcium channels [7], [61], [62] which is also discussed more extensively by Moreno and Vaca (2011) [63]. Additionally, it was shown that in non-mammal vertebrate such as Xenopus laevis initial response in commissural interneurons is also dependent on cyclic nucleotide-gated channel activity [64]. To fully understand and evaluate these links in mouse DRG axon response to Sema3A further investigations are needed.

Interestingly, after applying different Sema3A concentrations to DRG neurons, a steady and slow increase of intracellular calcium concentration occurred changing the plateau level of free cytoplasmic Ca^2+^ level. Similar elevation of intracellular calcium levels in response to Sema3A treatment was recently reported by Mitchell et al. (2012) [51]. We also showed that increase of [Ca^2+^]_i_ in response to Sema3A is a concentration dependent process, and thus can be important in understanding how axonal growth cones behave in Sema3A concentration gradient. We previously reported that axons growing downhill of a Sema3A gradient extended as good as axons growing downhill a control gradient not containing the inhibitory signal [65]. This intriguing result suggested the existence of adaptation mechanisms allowing growth cones to ignore inhibitory factors when experiencing decreasing concentration of the inhibitory guidance cue. Apart classical desensitization mechanism one can speculate that the elevated level of free cytoplasmic Ca^2+^ in response to Sema3A may define a threshold to pass in order to trigger growth cone collapse. Hence, any decreasing concentration of the factor may not be sufficient to overpass this newly established signaling gate thereby escaping from the inhibitory pressure of Sema3A. Besides this tempting hypothesis requiring difficult additional experiment modifying calcium levels of axons growing downhill Sema3A gradients the variable steady state of calcium level in response to Sema3A could explain why some laboratories [7], [8] claimed that Sema3A signaling is Ca^2+^ independent. In fact, Sema3A calcium signal is a long lasting event requiring seconds to be detected and is maintained for seconds, and is not as strong as calcium signals measured for other signals such as Slit-2, Netrin-1 or BDNF [43], [66], [51]. The long lasting duration in increase in Ca^2+^ concentration in the growth cones after Sema3A treatment suggest that the R-type Ca^2+^ channels activation is only part of the signaling cascade. For example, other parameters such as membrane potential or cGMP level as suggested by Nishiyama and colleagues [40] can play their role upstream of R-type Ca^2+^ channels activation. A multifactorial signaling is probably suiting any adaptation mechanisms needed to follow decreasing concentration of repellent. Hence, this long lasting reprogramming of the internal state of the growth cones throughout calcium intracellular level opens interesting perspective to clarify the signaling cascade of Sema3A that is still elusive.
